# Supplementary material for: Antibiotic and Heavy Metal Susceptibility of Non-Cholera Vibrio Isolated from Marine Sponges and Sea Urchins: Could They Pose a Potential Risk to Public Health?
Source: Antibiotics (Basel). 2021 Dec 20;10(12):1561. doi: 10.3390/antibiotics10121561 (PMC8698511; doi:10.3390/antibiotics10121561)
Supplement: Supplementary file 1 [file antibiotics-10-01561-s001.zip › antibiotics-1511586-SM-done.pdf]

Supplementary Materials

Antibiotic and heavy metal susceptibility of non-cholera *Vibrio* isolated from marine sponges and sea urchins: could they pose a potential risk to public health?

Wellington Felipe Costa, Marcia Giambiagi de-Marval, Marinella Silva Laport *

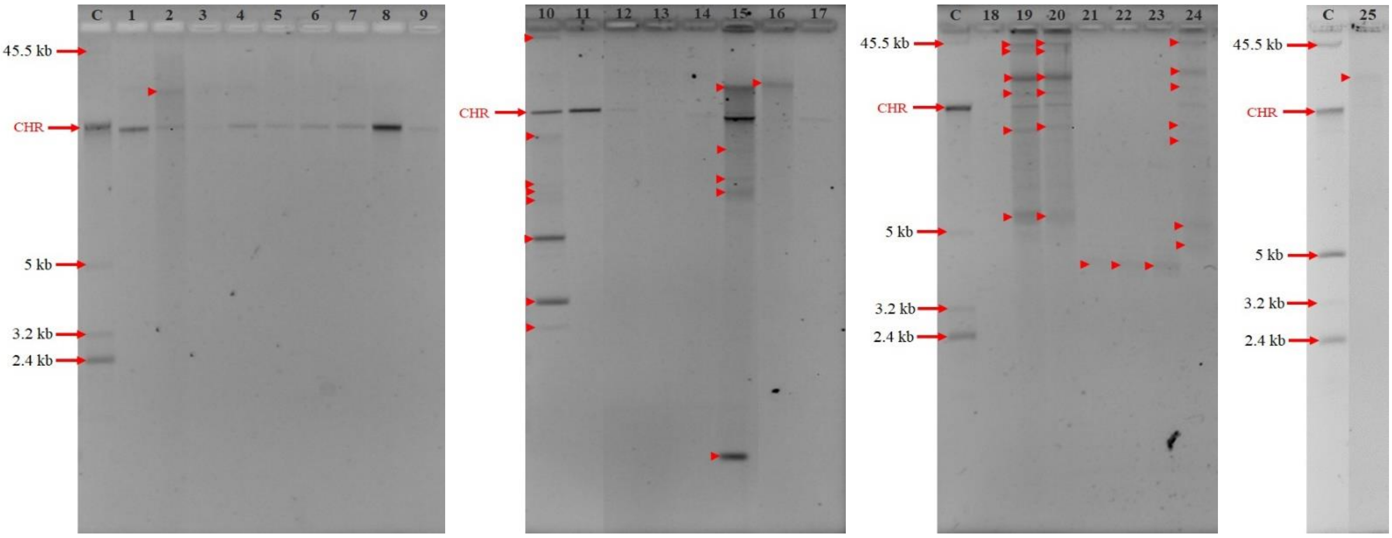

Figure S1. Plasmid profile of the *Vibrio* spp. strains. Plasmid DNA analyzed by 0.8% agarose gel electrophoresis. Lanes C: *K. pneumoniae* Kp13 (molecular size plasmids control); 1: 51BHI25; 2: 51BHI28; 3: 84BHI7b; 4: 51BHI31; 5: 51BHI31a; 6: 64BHI6; 7: 64BHI7; 8: 84BHI4; 9: 84BHI5; 10: 51BHI30; 11: 84BHI6; 12: 84BHI10; 13: 84B1:104; 14: 84MA3; 15: 84MA4; 16: HIM15.10^{-3}; 17: HEM9.10^{-3}; 18: ME7; 19: P1I-2; 20: P1I-8; 21: P3I-9; 22: P3I-12; 23: P3I-23; 24: P5I-6; 25: 84BHI7a. Fragmented chromosomal (CHR) and plasmid DNAs are indicated by arrows and arrow heads, on the left side.

Table S1. Identification, antimicrobial resistance profile, antimicrobial and heavy metal resistance genes, virulence genes and the plasmid profile of Vibrio spp. isolates from marine sponges (Brazil) and sea urchins (France).

Host	Strain	Accession Number[a]	BLASTn (%; Accession Number)[b]	MALDI-TOF identification (score)	Phenotypic Resistance	Resistance genes	Virulence genes	Plasmids[c]
	Vibrio sp. 51BHI25	KX148503	*Vibrio alginolyticus* (99.34; MH643615.1)	*Vibrio alginolyticus* (2.169)	CFE	-	-	-
Darwinella sp. 1 (marine sponge)	*Vibrio* sp. 51BHI28[d]	KX148504	*Vibrio alginolyticus* (99.10; MK988029.1)	*Vibrio alginolyticus* (1.758)	AMI, CFE, SXT[i]	-	-	1
	Vibrio sp. 51BHI30	KX148505	*Vibrio alginolyticus* (99.87; AP022865.1)	*Vibrio alginolyticus* (2.068)	-	-	-	8

	Vibrio sp. 51BHI31	KX148506	*Vibrio alginolyticus* (99.60; MK988029.1)	*Vibrio alginolyticus* (2.046)	AMC[i], CFE, SXT[i]	-	-	-
	Vibrio sp. 51BHI31a	KX148507	*Vibrio alginolyticus* (99.60; MK988029.1)	*Vibrio alginolyticus* (1.803)	CFE, ERY	-	-	-
Plakina cyanorosa (marine sponge)	*Vibrio* sp. 64BHI6	KX148484	*Vibrio alginolyticus* (97.09; MK988029.1)	*Vibrio alginolyticus* (2.169)	-	-	-	-
	Vibrio sp. 64BHI7	KX148485	*Vibrio alginolyticus* (98.94; MK102585.1)	*Vibrio alginolyticus* (2.054)	CFE	-	-	-
Darwinella sp. 2 (marine sponge)	*Vibrio* sp. 84BHI4	KX148486	*Vibrio alginolyticus* (98.50; MK452731.1)	*Vibrio alginolyticus* (2.165)	CFO[i], OFX[i]	-	-	-
	Vibrio sp. 84BHI5	KX148487	*Vibrio alginolyticus* (98.62; MH368400.1)	*Vibrio alginolyticus* (1.744)	CFE	-	-	-
	Vibrio sp. 84BHI6[d]	KX148488	*Vibrio alginolyticus* (97.73; MH368400.1)	*Vibrio alginolyticus* (1.803)	APS[i], CIP[i], ERY	-	-	-
	Vibrio sp. 84BHI7a	MH404096	*Vibrio proteolyticus* (94.39; GQ131641.1)	*Vibrio parahaemolyticus* (2.012)	CFE	-	-	1
	Vibrio sp. 84BHI7b	MH404095	*Vibrio azureus* (99.42; JQ665317.1)	*Vibrio mytili* (1.95)	AMI[i], CFE	-	-	-
	Vibrio sp. 84BHI10	KX148490	*Vibrio alginolyticus* (98.90; MN733131.1)	*Vibrio alginolyticus* (2.283)	APS[i], ERY	bla_{SHV}	-	-
	Vibrio sp. 84B1:104	KX148492	*Vibrio alginolyticus* (98.17; MH368400.1)	*Vibrio alginolyticus* (2.061)	ERY	-	-	-
	Vibrio sp. 84MA3	MF919340	*Vibrio alginolyticus* (99.17; MK988029.1)	*Vibrio alginolyticus* (2.133)	-	-	-	-
	Vibrio sp. 84MA4	MF919341	*Vibrio alginolyticus* (99.32; MN733127.1)	*Vibrio alginolyticus* (1.968)	AMI[h], APS[i], CFE, GEN[h], TOB[h]	-	-	5

Haliclona sp. (marine sponge)	*Vibrio* sp. HIM 15.10^{-3}	MG561916	*Vibrio harveyi* (100; CP035693.1)	*Vibrio harveyi* (2.254)	ERY	-	-	1
	Vibrio sp. HEM 9.10^{-3}	MH404093	*Vibrio neocaledonicus* (94.71; KX037098.1)	*Vibrio parahaemolyticus* (1.910)	ERY	-	-	-
	Vibrio sp. ME7	MH404094	*Vibrio coralliilyticus* (94.45; MH613238.1)	*Vibrio harveyi* (2.179)	ERY	-	*vvh*	-
Paracentrotus lividus (sea urchin)	*Vibrio* sp. P1I-2	KX898819	*Vibrio oceanisediminis* (98.29; NR_137371.1)	not reliable identification (1.438)	-	*cusB*	-	6
	Vibrionales P1I-8	KX898821	*Vibrio oceanisediminis* (99.26; NR_137371.1)	not reliable identification (1.425)	-	-	-	6
	Vibrio sp. P3I-9	KX898836	*Vibrio cyclitrophicus* (92.37; MK102602.1)	not reliable identification (1.323)	-	-	-	1
	Vibrionaceae P3I-12	KX898830	*Vibrio gigantis* (98.47; MH712999.1)	not reliable identification (1.242)	-	-	-	1
	Vibrio sp. P3I-23	KX898833	*Vibrio ichthyoenteri* (97.04; NR_117888.1)	not reliable identification (1.37)	-	-	-	1
	Vibrionales P5I-6	KX898861	*Vibrio oceanisediminis* (99.26; NR_137371.1)	not reliable identification (1.55)	-	-	-	7

([a]) GenBank accession numbers of *Vibrio* spp.' 16S rRNA sequences; ([b]) Strains isolation and 16S rRNA identification performed by Freitas-Silva et al. (2020) and Laport et al. (2018) studies; ([c]) number of plasmid forms; ([d]) multidrug-resistant *Vibrio* spp. strain; ([i]) intermediate; ([h]) heteroresistance; (-) negative test; AMC: amoxicillin-clavulanic acid; AMI: amikacin; APS: ampicillin-sulbactam; CFE: cephalexin; CFO: cefoxitin; CIP: ciprofloxacin; ERY: erythromycin; GEN: gentamicin; OFX: ofloxacin; SXT: trimethoprim/sulfamethoxazole; TOB: tobramycin.

Table S2. Primers used in this study.

Primer	Target	Sequence of Primers (5'-3')	Size (bp)	References
	β-lactams			
MTEMF<br>MTEMR	*bla*$_{TEM}$	5′-CCCTTATTCCCTTTYTTGCGG-3′*<br>5′-AACCAGCCAGCCWGAAGG-3′*	650	[36]
MSHVF<br>MSHVR	*bla*$_{SHV}$	5′-CTTGACCGCTGGGAAACGG-3′<br>5′-AGCACGGAGCGGATCAACGG-3′	200	
MGESF<br>MGESR	*bla*$_{GES}$	5′-AGCAGCTCAGATCGGTGTTG-3′<br>5′-CCGTGCTCAGGATGAGTTG-3′	750	
	aminoglycosides			
aac6-aph2a fw		5′-GCCAGAACATGAATTACACGAG-3′	611	[37]

aac6-aph2a rev	*aac(6′)-Ie-aph(2′)-Ia*	5′-CTGTTGTTGCATTTAGTCTTTCC-3′		
maac6-IbF	*aac(6′)-Ib*	5′-TTGCGATGCTCTATGAGTGGCTA-3′	482	[38]
maac6-IbR		5′-CTCGAATGCCTGGCGTGTTT-3′		
Fant	*ant(2″)-I*	5′-TGGGCGATCGATGCACGGCTRG-3′*	428	[39]
Rant		5′-AAAGCGGCACGCAAGACCTCMAC-3′*		
	macrolides			
MefC-F	*mef*(C)	5′-ATGGAAAACCGTAAATGGTT-3′	885	[40]
MefC-R		5′-TTAAATATTTTTGATTTTAC-3′		
MphG-F	*mph*(G)	5′-ATGAAAAATAGAGATATTCA-3′	1224	[40]
MphG-R		5′-CTACTCAACACCTAACTGTA-3′		
ermB-1	*ermB*	5′-CATTTAACGACGAAACTGGC-3′	425	[41]
ermB-2		5′-GGAACATCTGTGGTATGGCG-3′		
ereA-1	*ereA*	5′-AACACCCTGAACCCAAGGGACG-3′	420	[42]
ereA-2		5′-CTTCACATCCGGATTCGCTCGA-3′		
mphA-1	*mphA*	5′-AACTGTACGCACTTGC-3′	837	[42]
mphA-2		5′-GGTACTCTTCGTTACC-3′		
MF4a	*mefA*	5′-ACCGATTCTATCAGCAAAG-3′	940	[43]
MF6		5′-GGACCTGCCATTGGTGTG-3′		
	quinolones			
QnrAm-F	*qnrA*	5′-AGAGGATTTCTCACGCCAGG-3′	580	
QnrAm-R		5′-TGCCAGGCACAGATCTTGAC-3′		
QnrBm-F	*qnrB*	5′-GGMATHGAAATTCGCCACTG-3′*	264	
QnrBm-R		5′-TTTGCYGYYCGCCAGTCGAA-3′*		
QnrCm-F	*qnrC*	5′-GCGAATTTCCAAGGGGCAAA-3′	135	
QnrCm-R		5′-ACCCGTAATGTAAGCAGAGCAA-3′		[44]
QnrDm-F	*qnrD*	5′-AGGTGTAGCATGTATGGAAAAGC-3′	691	
QnrDm-R		5′-ACATTGGGGCATTAGGCGTT-3′		
QnrSm-F	*qnrS*	5′-GCAAGTTCATTGAACAGGGT-3′	428	
QnrSm-R		5′-TCTAAACCGTCGAGTTCGGCG-3′		
QnrVCm-F	*qnrVC*	5′-GAGYTKTATGGTTTAGAYCCTCG-3′*	71	
QnrVCm-R		5′-TGTTCYTGYTGCCACGARCA-3′*		
	heavy metals			
A1F	*merA*	5′-ACCATCGGCGGCACCTGCGT-3′		
A5R		5′-ACCATCGTCAGGTAGGGGACCAA-3′	1226	[45]
merA-F		5′-GTGCCGTCCAAGATCATGAT-3′	933	[46]
merA-R		5′-TAGCCYACRGTSGCSACYTG-3′*		
czcB-I	*czcB* and *cusB*	5′-ATGAAAAAAATCGCGCTT-3′	1200	[47]
czcB-II		5′- ATGCGCATGGGTAGCACT-3′		
cad1	*cadA*	5′-CAAAYTGYGCRGGHAARTTYGA-3′*	1058	[48]
cad2		5′-AACTAATGCACAAGGACA-3′		
pbrA-N	*pbrA*	5′-ATGAGCGAATGTGGCTCGAAG-3′	2400	[49]
pbrA-C		5′-TCATCGACGCAACAGCCTCAA-3′		
	Virulence genes			
toxR-F	*toxR*	5′-GTCTTCTGACGCAATCGTTG-3′	399	
toxR-R		5′-ATACGAGTGGTTGCTGTCATG-3′		[50]
tdh-F	*tdh*	5′-CCAAATACATTTTACTTGG-3′	251	

tdh-R		5′-GGTACTAAATGGCTGACATC-3′		
trh-F	*trh*	5′-GGCTCAAAATGGTTAAGCG-3′	250	
trh-R		5′-CATTTCCGCTCTCATATGC-3′		
VHF1	*vvh*	5′-ATCATGAATAAAACTATTACGTTACT-3′	1300	[25]
VHR1		5′-GAAAGGATGGTTTGACAAT-3′		
VP1409-F	*T6SS*	5′-TGTTGCTTTCTATTGCGAC-3′	869	[51]
VP1409-R		5′-CCATAACGACTTTTCTTTC-3′		
	Integrase Class 1			
intI1F	*intI1*	5′-GGCTTCGTGATGCCTGCTT-3′	146	[52]
intI1R		5′-CATTCCTGGCCGTGGTTCT-3′		

*: M = A or C; H = A or C or T; Y = C or T; K = T or G; R = A or G; S = G or C; W = A or T.

References

36. Campana, E.H.; Xavier, D.E.; Petrolini, F.V.; Cordeiro-Moura, J.R.; Araujo, M.R.; Gales, A.C. Carbapenem-resistant and cephalosporin-susceptible: a worrisome phenotype among *Pseudomonas aeruginosa* clinical isolates in Brazil. *Braz. J. Infect. Dis.* **2017**, *21*, 57-62, doi:10.1016/j.bjid.2016.10.008
37. Schiwon, K.; Arends, K.; Rogowski, K.M.; Fürch, S.; Prescha, K.; Sakinc, T.; et al. Comparison of antibiotic resistance, biofilm formation and conjugative transfer of *Staphylococcus* and *Enterococcus* isolates from International Space Station and Antarctic Research Station Concordia. *Microb. Ecol.* **2013**, *65*, 638-651, doi:10.1007/s00248-013-0193-4
38. Park, C.H.; Robicsek, A.; Jacoby, G.A.; Sahm, D.; Hooper, D.C. Prevalence in the United States of *aac(6')-Ib-cr* encoding a ciprofloxacin-modifying enzyme. *Antimicrob. Agents Chemother.* **2006**, *50*, 3953-3955, doi:10.1128/AAC.00915-06
39. Heuer, H.; Krögerrecklenfort, E.; Wellington, E.M.H.; Egan, S.; van Elsas, J.D.; van Overbeek, L.; et al. Gentamicin resistance genes in environmental bacteria: prevalence and transfer. *FEMS Microbiol. Ecol.* **2002**, *42*, 289-302, doi:10.1111/j.1574-6941.2002.tb01019.x
40. Nonaka, L.; Maruyama, F.; Suzuki, S.; Masuda, M. Novel macrolide-resistance genes, *mef*(C) and *mph*(G), carried by plasmids from *Vibrio* and *Photobacterium* isolated from sediment and seawater of a coastal aquaculture site. *Lett. Appl. Microbiol.* **2015**, *61*, 1-6, doi:10.1111/lam.12414
41. Jensen, L.B.; Frimodt-Møller, N.; Aarestrup, F.M. Presence of *erm* gene classes in gram-positive bacteria of animal and human origin in Denmark. *FEMS Microbiol. Lett.* **1999**, *170*, 151-158, doi:10.1111/j.1574-6968.1999.tb13368.x
42. Sutcliffe, J.; Grebe, T.; Kamradt, A.T.; Wondrack, L. Detection of erythromycin resistant determinants by PCR. *Antimicrob. Agents Chemother.* **1996**, *40*, 2562-2566, doi:10.1128/AAC.40.11.2562
43. Luna, V.A.; Cousin, S.-Jr.; Whittington, W.L.H.; Roberts, M.C. Identification of the conjugative *mef* gene in clinical *Acinetobacter junii* and *Neisseria gonorrhoeae* isolates. *Antimicrob. Agents Chemother.* **2000**, *44*, 2503-2506, doi:10.1128/aac.44.9.2503-2506.2000
44. Kraychete, G.B.; Botelho, L.A.B.; Campana, E.H.; Picão, R.C.; Bonelli, R.R. Updated multiplex PCR for detection of all six plasmid-mediated *qnr* gene families. *Antimicrob. Agents Chemother.* **2016**, *60*, 7524-7526, doi:10.1128/AAC.01447-16
45. De, J.; Ramaiah, N.; Vardanyan, L. Detoxification of toxic heavy metals by marine bacteria highly resistant to mercury. *Mar. Biotechnol. (NY)* **2008**, *10*, 471-477 doi:10.1007/s10126-008-9083-z
46. Deredjian, A.; Colinon, C.; Brothier, E.; Favre-Bonté, S.; Cournoyer, B.; Nazaret, S. Antibiotic and metal resistance among hospital and outdoor strain of *Pseudomonas aeruginosa*. *Res. Microbiol.* **2011**, *162*, 689-700, doi:10.1016/j.resmic.2011.06.007
47. Khan, Z.; Nisar, M.A.; Hussain, S.Z.; Arshad, M.N.; Rehman, A. Cadmium resistance mechanism in *Escherichia coli* P4 and its potential use to bioremediate environmental cadmium. *Appl. Microbiol. Biotechnol.* **2015**, *99*, 10745-10757, doi:10.1007/s00253-015-6901-x
48. Oger, C.; Bertheb, T.; Quilleta, L.; Barraya, S.; Chiffoleauc, J.F.; Petit, F. Estimation of the abundance of the cadmium resistance gene *cadA* in microbial communities in polluted estuary water. *Res. Microbiol.* **2001**, *152*, 671-678, doi:10.1016/s0923-2508(01)01246-3
49. Borremans, B.; Hobman, J.L.; Provoost, A.; Brown, N.L.; van-Der-Lelie, D. Cloning and functional analysis of the *pbr* lead resistance determinant of *Ralstonia metallidurans* CH34. *J. Bacteriol.* **2001**, *183*, 5651-5658, doi:10.1128/JB.183.19.5651-5658.2001

50. Guin, S.; Saravanan, M.; Chowdhury, G.; Pazhani, G.P.; Ramamurthy, T.; Das, S.C. Pathogenic *Vibrio parahaemolyticus* indiarrhoeal patients, fish and aquatic environments and their potential for inter-source transmission. *Heliyon* **2019**, *5*, 01743, doi:10.1016/j.heliyon.2019.e01743
51. Chao, G.; Jiao, X.; Zhou, X. et al. Distribution of genes encoding four pathogenicity islands (VPaIs), T6SS, biofilm, and type I pilus in food and clinical strains of *Vibrio parahaemolyticus* in China. *Foodborne Pathog. Dis.* **2010**, *7*, 649-658, doi:10.1089/fpd.2009.0441
52. Jiang, H.; Yu, T.; Yang, Y.; Yu, S.; Wu, J.; Lin, R.; Li, Y.; Fang, J.; Zhu, C. Co-occurrence of antibiotic and heavy metal resistance and sequence type diversity of *Vibrio parahaemolyticus* isolated from *Penaeus vannamei* at freshwater farms, seawater farms, and markets in Zhejiang Province, China. *Front. Microbiol.* **2020**, *11*, 1294, doi:10.3389/fmicb.2020.01294
